# Editorial: Radiotherapy and commensal microbiome

**DOI:** 10.3389/fmicb.2024.1484953

**Published:** 2024-10-07

**Authors:** Sonia M. Rodrigues Oliveira, Veeranoot Nissapatorn, Maria De Lourdes Pereira

**Affiliations:** ^1^Universidade Católica Portuguesa, Faculty of Dental Medicine (FMD), Center for Interdisciplinary Research in Health, Viseu, Portugal; ^2^CICECO-Aveiro Institute of Materials, University of Aveiro, Aveiro, Portugal; ^3^School of Allied Health Sciences and World Union for Herbal Drug Discovery, Walailak University, Nakhon Si Thammarat, Thailand; ^4^Department of Medical Sciences, University of Aveiro, Aveiro, Portugal

**Keywords:** radiotherapy, radiation, microbiota, radiotherapy resistance, radiation toxicity, commensal microbiome, probiotics, diet and nutrition supply

Radiation therapy (Radiotherapy) is one of the most important and used methods to fight cancer by killing cancer cells, shrinking tumors, and relieving symptoms due to the precise application of high doses of radiation. At low doses, radiation is used in x-rays, but in high doses is a cornerstone cancer treatment, which is increasingly recognized for its complex interactions with the human microbiome. Recent advances highlight the bidirectional influence between radiation therapy and commensal microbiota, suggesting potential therapeutic implications. This Research Topic, “*Radiotherapy and commensal microbiome*,” underscores some of these interactions, providing insights into the mechanisms through which radiation modulates microbiota responses and how radiotherapy affects microbial communities.

The multifaceted relationship between radiotherapy and the microbiome is reflected in the diverse array of studies we received, ultimately publishing six impactful articles. These contributions highlight the importance of understanding microbiome dynamics in the context of radiotherapy and offer potential pathways to enhance therapeutic efficacy and mitigate adverse effects.

One of the notable studies, “*Shelf life and simulated gastrointestinal tract survival of selected commercial probiotics during a simulated round-trip journey to Mars*” (Fajardo-Cavazos and Nicholson), investigates the resilience of probiotics in extreme conditions. While not directly linked to radiotherapy, this research provides foundational knowledge on probiotic stability, which is crucial for developing microbiome-targeted interventions in cancer patients undergoing radiotherapy. Understanding how probiotics can survive and function in hostile environments can inform strategies to maintain or restore gut health during and after radiotherapy.

In “*Gut microbiota characteristics are associated with severity of acute radiation-induced esophagitis*” (Lin et al.), authors explore the correlation between gut microbiota composition and the severity of radiation-induced esophagitis. This study underscores the potential for gut microbiota profiling to predict and manage radiotherapy-related complications. By identifying specific microbial signatures associated with adverse outcomes, this research paves the way for personalized microbiome-modulating therapies to enhance patient resilience to radiotherapy.

Another significant contribution, “*Metformin alleviates irradiation-induced intestinal injury by activation of FXR in intestinal epithelia*” (Yang et al.), examines the protective effects of metformin on the gut epithelium during irradiation. The study reveals that metformin's activation of the FXR pathway can mitigate intestinal damage, highlighting a novel therapeutic avenue. This finding not only enhances our understanding of metformin's radioprotective properties but also suggests a potential role for microbiome modulation in safeguarding gut integrity during cancer treatment.

The interplay between intratumoral microbiota and treatment response is explored in “*Interaction between intratumoral microbiota and tumor mediates the response of neoadjuvant therapy for rectal cancer*” (Sun et al.). This Research Topic demonstrates that intratumoral bacteria can influence the efficacy of neoadjuvant therapy, suggesting that microbiome composition within tumors could be a determinant of treatment success. The study supports integrating microbiome analysis into oncological protocols to optimize therapeutic outcomes.

“*The role of oral microbiota in cancer*” (Lan et al.) delves into the significance of oral microbiota in cancer development and progression. The authors discuss how dysbiosis in the oral cavity can contribute to oncogenesis and influence response to treatment, including radiotherapy. This review emphasizes the need for holistic microbiome research encompassing various body sites to fully grasp the microbiome's role in cancer biology.

Lastly, “*Investigating the effects of radiation, T cell depletion, and bone marrow transplantation on murine gut microbiota*” (Kreisinger et al.), provides valuable insights into how different cancer treatments impact gut microbiota. This study suggests that combined treatment modalities can significantly alter microbial communities, which may affect patient outcomes. Understanding these changes is critical for developing comprehensive care strategies that incorporate microbiome health.

Collectively, these articles feature the intricate relationship between radiotherapy and the commensal microbiome. They highlight the necessity of incorporating microbiome research into clinical practice to optimize cancer treatments. In the future, continued exploration of microbiome dynamics will be essential for developing innovative, personalized, therapeutic strategies that enhance the efficacy of radiotherapy while minimizing its adverse effects.

